# Tuberculosis (TB) Aftermath: study protocol for a hybrid type I effectiveness-implementation non-inferiority randomized trial in India comparing two active case finding (ACF) strategies among individuals treated for TB and their household contacts

**DOI:** 10.1186/s13063-022-06503-6

**Published:** 2022-08-05

**Authors:** Samyra R. Cox, Abhay Kadam, Sachin Atre, Akshay N. Gupte, Hojoon Sohn, Nikhil Gupte, Trupti Sawant, Vishal Mhadeshwar, Ryan Thompson, Emily Kendall, Christopher Hoffmann, Nishi Suryavanshi, Deanna Kerrigan, Srikanth Tripathy, Arjunlal Kakrani, Madhusudan S. Barthwal, Vidya Mave, Jonathan E. Golub, on behalf of the TB Aftermath study team, Sunil Ambike, Jayshri Jagtap, Pallavi Kadam, Shankar Jadhav, Anita Mahajan, Yogesh Bhosale, Vaibhavi Bodhe, Gautami Walunj, Sachin Rathod, Akshay Bhalchim

**Affiliations:** 1 Johns Hopkins Bloomberg School of Public Health, 615 N Wolfe St, Baltimore, MD 21205 USA; 2 Johns Hopkins School of Medicine, 600 N Wolfe St, Baltimore, MD 21287 USA; 3 Johns Hopkins India, G-4 & G-5, PHOENIX Building, OPP. to Residency Club, Pune, Maharashtra 411001 India; 4 Dr. D.Y. Patil Medical College, Hospital and Research Centre, Dr. D.Y. Patil Vidyapeeth, Sant Tukaram Nagar, Pimpri Colony, Pimpri-Chinchwad, Maharashtra 411018 India; 5 Seoul National University College of Medicine, 103 Daehak-ro, Jongno-gu Seoul, 03080 Republic of Korea; 6 George Washington University, 2121 I St NW, Washington, D.C., 20052 USA

**Keywords:** Tuberculosis, Recurrence, Active case finding, India, Hybrid effectiveness-implementation trial

## Abstract

**Background:**

Approximately 7% of all reported tuberculosis (TB) cases each year are recurrent, occurring among people who have had TB in the recent or distant past. TB recurrence is particularly common in India, which has the largest TB burden worldwide. Although patients recently treated for TB are at high risk of developing TB again, evidence around effective active case finding (ACF) strategies in this population is scarce. We will conduct a hybrid type I effectiveness-implementation non-inferiority randomized trial to compare the effectiveness, cost-effectiveness, and feasibility of two ACF strategies among individuals who have completed TB treatment and their household contacts (HHCs).

**Methods:**

We will enroll 1076 adults (≥ 18 years) who have completed TB treatment at a public TB unit (TU) in Pune, India, along with their HHCs (averaging two per patient, *n* = 2152). Participants will undergo symptom-based ACF by existing healthcare workers (HCWs) at 6-month intervals and will be randomized to either home-based ACF (HACF) or telephonic ACF (TACF). Symptomatic participants will undergo microbiologic testing through the program. Asymptomatic HHCs will be referred for TB preventive treatment (TPT) per national guidelines. The primary outcome is rate per 100 person-years of people diagnosed with new or recurrent TB by study arm, within 12 months following treatment completion. The secondary outcome is proportion of HHCs < 6 years, by study arm, initiated on TPT after ruling out TB disease. Study staff will collect socio-demographic and clinical data to identify risk factors for TB recurrence and will measure post-TB lung impairment. In both arms, an 18-month “mop-up” visit will be conducted to ascertain outcomes. We will use the RE-AIM framework to characterize implementation processes and explore acceptability through in-depth interviews with index patients, HHCs and HCWs (*n* = 100). Cost-effectiveness will be assessed by calculating the incremental cost per TB case detected within 12 months and projected for disability-adjusted life years averted based on modeled estimates of morbidity, mortality, and time with infectious TB.

**Discussion:**

This novel trial will guide India’s scale-up of post-treatment ACF and provide an evidence base for designing strategies to detect recurrent and new TB in other high burden settings.

**Trial registration:**

NCT04333485, registered April 3, 2020.

CTRI/2020/05/025059 [Clinical Trials Registry of India], registered May 6 2020.

**Supplementary Information:**

The online version contains supplementary material available at 10.1186/s13063-022-06503-6.

## Administrative information

Note: the numbers in curly brackets in this protocol refer to SPIRIT checklist item numbers. The order of the items has been modified to group similar items (see http://www.equator-network.org/reporting-guidelines/spirit-2013-statement-defining-standard-protocol-items-for-clinical-trials/).

**Table:** 

Title {1}	Tuberculosis (TB) Aftermath: study protocol for a hybrid type I effectiveness-implementation non-inferiority randomized trial in India comparing two active case finding (ACF) strategies among individuals treated for TB and their household contacts
Trial registration {2a and 2b}.	NCT04333485 [ClinicalTrials.gov], Registered April 3, 2020 CTRI/2020/05/025059 [Clinical Trials Registry of India], registered May 6 2020.
Protocol version {3}	Version 1.3, January 28, 2022
Funding {4}	National Institutes of Health (NIH), National Institute of Allergy and Infectious Diseases (NIAID), R01AI143748
Author details {5a}	1. Johns Hopkins Bloomberg School of Public Health, Baltimore, MD, USA 2. Johns Hopkins School of Medicine, Baltimore, MD, USA 3. Johns Hopkins India, Pune, Maharashtra, India 4. George Washington University, Washington, D.C., USA 5. Dr. D.Y. Patil Medical College, Hospital and Research Centre, Dr. D.Y. Patil Vidyapeeth, Pune, Maharashtra, India 6. Seoul National University College of Medicine, Seoul, Republic of Korea
Name and contact information for the trial sponsor {5b}.	Karen A Lacourciere, NIH/NIAID Program Officer, lacourcierek@niaid.nih.gov
Role of sponsor {5c}	The sponsor had no role in the study design or writing of this manuscript.

## Introduction

### Background and rationale {6a}

#### Disease burden

Over four million people who developed tuberculosis (TB) worldwide in 2020 were never diagnosed and linked to care. The missing four million are among an estimated 9.9 million new or recurrent cases, of which 1.5 million died. India has the largest TB burden in the world, with over 2.5 million cases and approximately half a million deaths each year [1]. Further, it is estimated that nearly a quarter of the world’s missing cases are in India. TB control is a priority for the Indian government and they have set an ambitious target to eliminate the disease by 2025 [2]. Despite a 24% reduction in incidence over the past decade [3], an increase in cases is expected due to pandemic-related setbacks [1, 4]. Specifically, models of interruptions in TB services due to COVID-19 project an increase of 182,000 TB cases and 83,600 TB deaths in India between 2020 and 2025 [5]. As part of their response, the Indian Government has released modified recommendations for detecting and treating TB in the context of COVID-19 [6, 7]. Scale-up of TB preventive treatment (TPT) is also being prioritized and is now recommended for all household contacts (HHCs) in India after ruling out TB disease [8]. However, to tackle India’s TB epidemic, optimized active case finding (ACF) strategies that specifically target high-risk populations will also be needed.

#### Recurrent TB

Individuals who recently completed TB treatment are a high-risk group who should be targeted for ACF. Recurrent TB arises from either endogenous reactivation (relapse) or exogenous reinfection and people who have been treated for TB are at risk for both [9–11]. Despite a rise in treatment success rates from 69 to 86% over the past two decades [1, 12], recurrent TB still accounts for approximately seven percent of reported TB cases worldwide [13, 14]. In addition, persistent social or biological risk factors among individuals who have had TB once place them at increased risk of getting reinfected and developing TB again [15, 16]. Recurrent TB poses a threat to control programs, as it is associated with lower cure rates than new TB [17], drug resistance [18, 19], and expensive retreatment regimens which can further exhaust national budgets for TB control [20].

In India, TB recurrence is particularly common; a meta-analysis of seven studies documented an overall recurrence rate of approximately 10% [21]. A more recent study showed that 13% of cured and treatment completed patients developed recurrent TB within 1 year of treatment end, for a weighted recurrence rate of 12.7/100 person-years [22]—a TB incidence rate that is over 60 times the national average. That same review concluded that routine follow-up of all TB patients for 1 year after treatment completion may be an efficient mechanism for identifying new cases of TB. Recent TB patients are also a point of contact with another group at elevated risk for TB: their household contacts. In a systematic review and meta-analysis, HHCs of patients with TB in low- and middle-income settings had a 3% TB prevalence at the time of initial contact investigation and a TB incidence of 1500 per 100,000 in the subsequent year [23].

#### Rationale for active case finding among treated TB patients

Directing surveillance resources towards “adequately” treated patients at high risk for recurrent disease is supported by the finding that such patients experience TB risks comparable to other subgroups already targeted by ACF. Although it is clear that the burden of recurrent disease among recently-treated patients is high, evidence for (or against) the effectiveness of ACF in this population is scarce. To date, there are no published ACF trials targeting treated TB patients and their HHC. As a result, the 2021 World Health Organization (WHO) guidelines for systematic screening for TB disease could make only a conditional recommendation that individuals previously treated for TB should be screened in high-incidence settings (≥ 100 cases per 100,000 people) [24]. However, the potential impact of ACF is high. A recent modeling study in South Africa suggests that ACF for recurrent TB would reduce average duration of TB from 9.7 to 5 months among previously-treated TB patients, with a tremendous potential impact on transmission [25]. From a feasibility standpoint, people treated for TB are an ideal population for ACF as they were recently traceable for at least 6 months of TB treatment, making them easier to locate.

#### Which patients develop recurrent TB?

Identifying TB patients likely to develop recurrent disease could help target case-finding efforts and addressing modifiable risk factors could reduce the risk of recurrence. Prior studies have found an association between poor adherence to TB treatment and early recurrence suggestive of relapse [26, 27]. HIV is also a well-recognized risk factor for TB recurrence [10, 28, 29]. Diabetes is associated with a threefold higher risk of TB disease [30] and may be associated with recurrence as well [31, 32]. Alcohol consumption and exposure to tobacco smoke have both been shown to correlate with recurrent disease [28, 33–36]. Similarly, there is evidence that indoor air pollution increases risk of TB disease [37, 38]. Clinically, the pulmonary TB and lung impairment dynamic may be a vicious cycle; treated TB patients with pulmonary sequelae may be at higher risk of chronic lung diseases and subsequently recurrent TB [39]. Finally, socio-economic status and poverty are strongly linked to TB and likely impact risk of recurrence [40–42].

#### Understanding implementation of ACF among treated TB patients

Following former patients for recurrent TB disease presents challenges of clinic-to-community continuity and coordination. Notable examples of using community health workers for ACF in TB programs suggest that this model can be successful [43–45]. Despite these successes, a clear understanding of aspects of optimal implementation is lacking. As a result, multiple examples of under-delivery stand in contrast to the successful use of integrated clinic-community programs [46–49]. Through qualitative and quantitative measures, implementation science provides tools to describe HCW and patient perceptions, management, and role concordance, all of which are used to develop systematic management and implementation strategies. These assessments are vital to guide future implementation.

#### Rationale for comparing home-based vs. telephonic ACF

India’s National TB Elimination Program (NTEP) is dedicated to rolling out a strategy for ACF among treated TB patients; however, they have not yet defined the strategy they will utilize. In Maharashtra, one of the highest TB burden states in India [50], NTEP policymakers and implementers are enthusiastic about studying the effectiveness of telephonic active case finding (TACF) and home-based active case finding (HACF). HACF strategies have been successful at identifying high proportions of TB among HHCs in high incidence settings [51–53]. However, HACF may be resource-intensive and difficult to scale. Thus, a TACF strategy should be tested. Our hypothesis is that those who need screening will be identified through TACF at a fraction of the cost of HACF. The diagnostic yield is anticipated to be high with both strategies, but differences in proportions of patients and households (HHs) screened, timeliness of the screening and associated costs are not known. TB Aftermath will help determine the most effective and cost-effective of these two post-treatment ACF strategies. Our hybrid type 1 effectiveness-implementation design includes implementation assessments of these strategies, at least one of which will be recommended for rollout in India. This novel trial aligns with the current NTEP strategy and has the potential to guide case detection strategies in India and other high TB burden settings.

#### Objectives {7}

The overall purpose of TB aftermath is to compare the effectiveness, cost-effectiveness, and feasibility of two ACF strategies for detecting recurrent TB and provide evidence needed to implement and scale up the preferred ACF strategy. Our specific objectives are:

1. To conduct a non-inferiority randomized trial to measure the comparative effectiveness of two potentially implementable ACF strategies within the NTEP, conducted by existing NTEP healthcare workers (HCWs): (i) home-based ACF (HACF) and (ii) telephonic ACF (TACF)
2. To characterize implementation processes of the ACF strategies using the RE-AIM framework to inform their future scale-up and sustainability [54]
3. To model the impact and cost-effectiveness of the ACF strategies evaluated in the trial, and of potential alternative strategies for the targeting and timing of those strategies
4. To measure the association of the severity, chronicity, and progression of post-TB lung impairment with recurrent TB disease

#### Trial design {8}

TB Aftermath is a hybrid type I effectiveness-implementation non-inferiority trial with individual randomization. A hybrid type I study is defined by Curran et al. as “testing a clinical intervention while gathering information on its delivery during the effectiveness trial and/or on its potential for implementation in a real-word situation.” [55] The participant allocation ratio between the HACF arm and the TACF arm is 1:1.

## Methods: participants, interventions, and outcomes

### Study setting {9}

The study will be conducted in three rural and three urban TB Units (TUs) of the NTEP in Pune district, Maharashtra, India. Each of the six participating TUs has the capacity to diagnose TB, initiate treatment, monitor TB treatment outcomes, and serve as directly observed therapy (DOT) centers. Each TU serves a population of approximately 300,000 to 500,000 people. Current responsibilities of existing TU HCWs include diagnosing TB suspects, linking newly diagnosed patients to TB care, visiting HHs within 7 days of index patient treatment initiation, screening HHCs for TB symptoms, referring potentially eligible HHCs for TPT, providing counseling on infection control, ensuring treatment adherence, and finding patients that are lost-to-follow-up. These responsibilities may be distributed among several positions. Urban TUs are usually staffed by a senior TB treatment supervisor (STS), senior TB laboratory supervisor (STLS), lab technician, and a TB home visitor, whereas rural TUs are only staffed by STS and STLS. For this trial, the role of selected NTEP HCWs will be extended to include additional follow-up on patients and HHs beyond treatment.

### Eligibility criteria {10}

The following criteria applies to enrolment of index patients with TB:

Inclusion criteria:

- Adults (≥ 18 years of age) who are registered at one of the participating TUs as TB treatment completed or cured (regardless of type of TB or duration of treatment)
- Confirmed treatment completion status by the referring medical officer of the participating TU
- Date of treatment completion within 60 days of date of study enrolment
- Ability and willingness of participant or legal guardian/representative to provide informed consent to participate in the HACF or TACF arm

Exclusion criteria:

- Completed TB treatment at a private sector clinic or TU outside of the study (final visit not registered at one of the participating TUs)
- Actively on TB treatment

There will be no exclusion criteria according to sex/gender, HIV status, type of TB, or racial/ethnic group. For enrolment of HHCs, all individuals, regardless of age, who are able and willing to provide informed consent are eligible to participate.

### Who will take informed consent? {26a}

Study counselors will obtain written informed consent from potential trial participants in person. After discussing study procedures, risk, and benefits in the local language (Marathi or Hindi), the study counselor will test the individual’s understanding. Individuals will be given time to ask questions, reflect, and discuss with family before being invited to provide consent. Eligible index patients will be consented at end of treatment completion to participate for 18 months. HHCs will be consented at the first home visit. For HHCs who are < 18 years or individuals unable to consent for themselves, informed consent will be sought from a legal guardian/representative and additionally, per national guidelines, oral or written assent will be sought from minors.

### Additional consent provisions for collection and use of participant data and biological specimens {26b}

N/A. Biological specimens will not be stored.

### Interventions

#### Explanation for the choice of comparators {6b}

In order to provide evidence for an effective and scalable strategy for detecting recurrent TB among index patients and new cases among HHCs, we will compare two ACF strategies: (i) ACF by home-based symptom screen (HACF) and sputum collection at the TU for symptomatic HHCs and (ii) ACF by telephonic symptom screen (TACF) followed by sputum collection at the TU for symptomatic HHCs.

#### Intervention description {11a}

In both arms, ACF will be conducted by existing NTEP HCWs at 6 and 12 months after the index patient completes TB treatment. Based on the WHO and Government of India guidelines, the HCW will screen for any symptom of TB, including cough for ≥ 2 weeks, hemoptysis, fever, night sweats, weight loss, or lymph node swelling to screen for extrapulmonary TB [24, 56]. Participants with any symptom of TB will be asked to visit the TU to provide a spot sputum specimen. As per routine programmatic conditions, sputa will undergo Xpert testing (or available microbiologic testing) and results will be returned to the patient.

#### Criteria for discontinuing or modifying allocated interventions {11b}

Participants may withdraw from the study at any time due to personal reasons. In such cases, the participant’s record while in the study will be maintained along with a progress note describing reason for withdrawal. A participant’s allocated arm may not be modified at any time during the study.

#### Strategies to improve adherence to interventions {11c}

To improve adherence to the intervention procedures, HCWs at the participating TUs will be trained in how and when to conduct HACF and TACF. Refresher trainings will be offered periodically. Before each scheduled follow-up, the study staff will provide reminders to NTEP HCWs, and the number of reminders will be tracked. Challenges faced by HCWs will be documented in follow-up logs and regularly assessed to improve strategies. An internal monitor will review procedures on an annual basis, including assessing adherence to the intervention procedures.

#### Relevant concomitant care permitted or prohibited during the trial {11d}

Participants may not be enrolled in another clinical study involving follow-up time points that conflict with TB Aftermath. However, clinical care may be sought at the participant’s discretion during the study period. Participants who report symptoms suggestive of TB disease will be referred to the TU for evaluation. Likewise, asymptomatic HHCs who are identified as potentially eligible for TPT per NTEP guidelines but who have yet to access services will be referred for further evaluation. Index patients newly diagnosed with diabetes at entry, and any participant with lung impairment will also be referred for appropriate care.

#### Provisions for post-trial care {30}

N/A. There is no more than minimal risk to participants associated with study procedures. Thus, post-trial care will not be required.

### Outcomes {12}

#### Primary outcome

The rate per 100 person-years of people diagnosed with new or recurrent TB by study arm, within 12 months following index TB patient’s treatment completion date. TB disease will be diagnosed as microbiologically confirmed (positive acid fast bacilli smear or positive Xpert® MTB/RIF assays or positive culture) or clinically diagnosed (initiated on TB treatment with no microbiological confirmation) [57]. Primary analyses will be based on microbiologically confirmed TB cases among index patients (recurrent TB) or HHCs (new TB), and all analyses will be repeated including clinically diagnosed TB.

#### Secondary outcome

The secondary outcome will be proportion of eligible HHCs < 6 years of age, by study arm, initiated on TPT after ruling out active TB disease. NTEP guidelines include initiation of TPT in children < 6 years of age who are contacts of a patient with TB. At enrolment, participants will report names of HHCs < 6 years of age and their history of TPT. Children who have not yet initiated TPT will be the denominator and children who initiate TPT following index patient enrolment will be the numerator. We will also measure if proportion initiating TPT differs by study arm.

### Participant timeline {13}

The TB Aftermath schedule of evaluations, including responsible personnel, is described for index patients and HHCs in Table 1.

**Table 1:** TB aftermath schedule of evaluations

Activity/evaluation	Time point				Administration	
	Entry	+M6	+M12	+M18 (Mop-up)	NTEP HCW	Study staff
**Index patients**						
Informed consent, enrolment and randomization	X					X
Socio-demographic and clinical questionnaires	X			X		X
Household geo-coding	X					X
Biomass fuel, smoking and alcohol exposure questionnaires	X			X		X
Point-of-care HbA1c test	X					X
Lung health assessments	X	X^a^	X^a^	X		X
Modified WHO survey for TB patient costs	X	X		X		X
TB symptom questionnaire	X	X	X	X	X	
TB event questionnaire		Any time TB case is identified				X
**Household contacts**						
Informed consent and enrolment^b^		X	X	X		X
Clinical questionnaire		X^a^		X		X
Lung health assessments		X^a^		X^a^		X
TB symptom questionnaire		X	X	X	X	
TB event questionnaire		Any time TB case is identified				X

^a^Only among HACF arm participants

^b^Study data will not be collected directly from HHCs unless a home visit is conducted and they are consented

*Abbreviations*: *TB* tuberculosis, *WHO* World Health Organization, *HACF* home-based active case finding

**Fig. 1:**
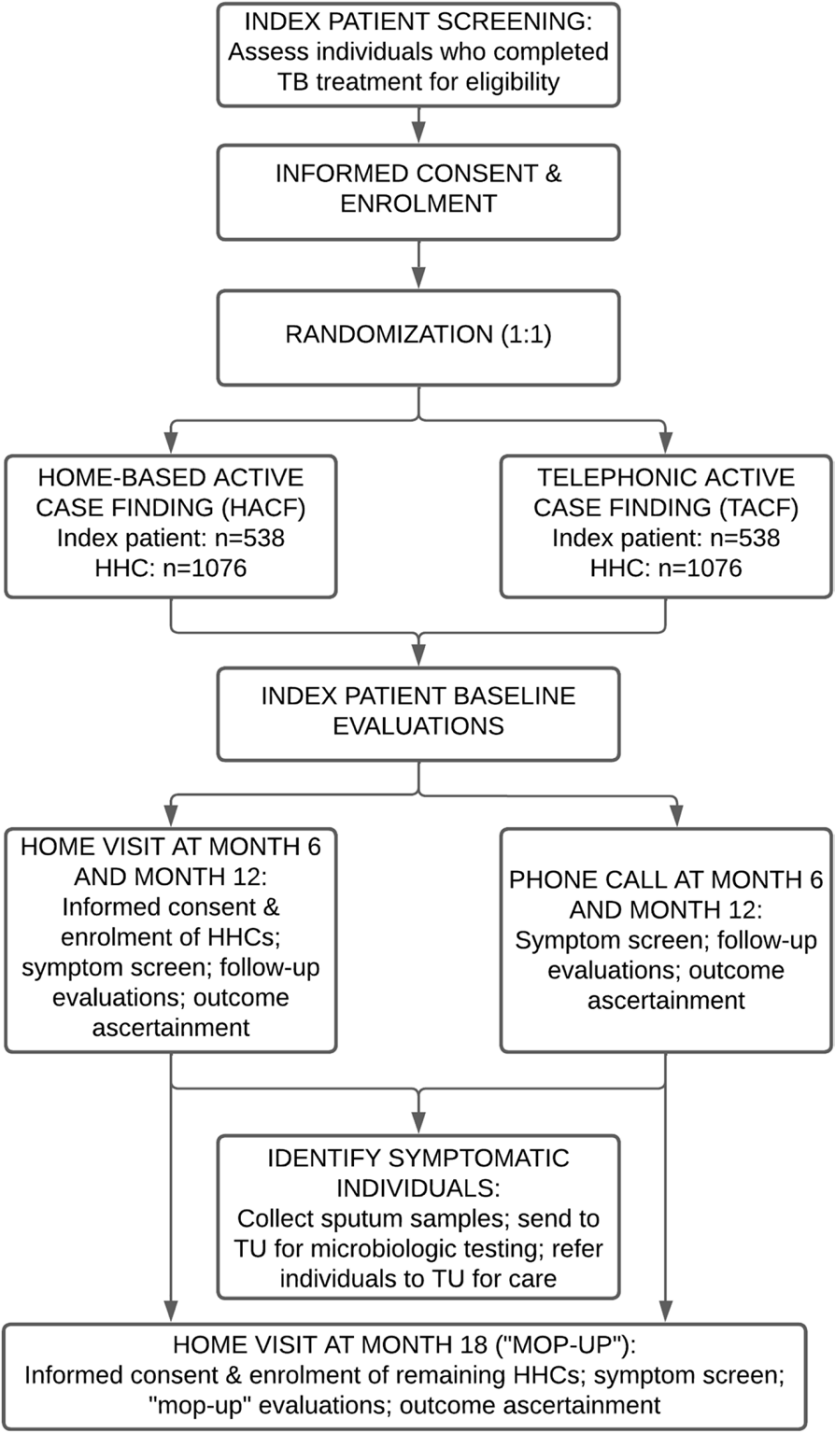
TB Aftermath study flow diagram. TB, tuberculosis; HACF, home-based active case finding; TACF, telephonic active case finding; HHC, household contact; TU, TB unit

The TB Aftermath study flow diagram is depicted in Fig. 1.

### Sample size {14}

The sample size for index patients (*n* = 1076) includes 538 in the HACF arm and 538 in the TACF arm (1:1 ratio). On average, we will enroll two HHCs per index patient (*n* = 2152). The total estimated sample size for index patients and HHCs is 3228 individuals.

Data from relevant studies suggest that 10–13% of index patients will develop recurrent TB in each arm [21, 22] and an additional 1–2% of HHCs will develop TB during the study period [58]. Assuming a rate of 12 TB cases per 100 person-years among index patients and 1 TB case per 100 person-years among HHCs, we are powered at 90% to determine that the TACF arm is non-inferior to the HACF arm with a non-inferiority interval of 1.7 per 100 person-years and a sample size of 1076 index patients and two HHCs per index patient (*n* = 2152). This sample size for the primary outcome is adjusted for 10% projected losses to follow-up. For the secondary outcome, we anticipate that 10% (215/2152) of HHCs will be children < 6 years of age. Data from Pune suggest that approximately 30% of child contacts will have been screened for TB and either initiated on TB therapy or TPT [59]. Thus, we anticipate 70 eligible children in each study arm and hypothesize > 60% to be screened and initiated on TB therapy or TPT in the HACF arm and less than 20% in the TACF arm. We will have at least 90% power to see a difference of 60% vs 20% and 80% power to see a difference of 50% vs 25%.

For objective 2, we will conduct a total of 40 in-depth interviews with index patients (20 with and 20 without recurrent TB) and one HHC for each patient (40 total). We will also interview 20 HCWs to achieve a total sample size of 100. We will use purposive sampling to obtain a maximum variation sample by study arm, as well as gender and age group for index patients, HHCs, and a range of HCW types and levels.

### Recruitment {15}

On a monthly basis, study staff will review NTEP registers to identify all potentially eligible participants nearing the end of their TB treatment at participating TUs. Patients for whom treatment was extended will also be tracked. All potential trial participants will be contacted and screened for enrolment with 60 days of treatment completion. For both index TB cases and HHCs who do not agree to participate, reasons will be documented and discussed by study staff. Accrual reports will be reviewed regularly and additional TUs will be added if required to achieve target sample size.

### Assignment of interventions: allocation

#### Sequence generation {16a}

Trial randomization will be computer-generated, using permuted blocks of equal size with balancing by participating TU.

#### Concealment mechanism {16b}

The computer-generated allocation (indicating HACF or TACF arm) will be blindly placed in sealed envelopes by data managers. Envelopes will be labeled with the randomization number and stored in numeric order at the TU. After the participant provides informed consent, study counselors will select the next sealed envelope.

#### Implementation {16c}

The lead data analyst will run the complete computer-generated allocation list prior to the first enrolment (with 10–15% extra for anticipated losses to follow-up). For each participant, the study counselor will obtain informed consent prior to opening the sealed envelope with allocation number (indicating HACF or TACF arm).

### Assignment of interventions: blinding

#### Who will be blinded {17a}

This is an open-label trial in which both the participants and study staff will be aware of intervention arm after randomization. However, in an effort to reduce potential bias, principal investigators (PIs) and the co-investigators who will be analyzing results will be blinded as to intervention arm.

#### Procedure for unblinding if needed {17b}

N/A. The trial is open-label, and unblinding PIs and co-investigators will not be required.

## Data collection and management

### Plans for assessment and collection of outcomes {18a}

#### Objective 1: data collection

##### HACF arm

The NTEP HCW will visit participant homes at 6 and 12 months post-treatment completion to screen the index patient and HHCs for TB. All HH members who screen positive will be asked to visit the TU to provide a spot sputum specimen for microbiological testing at the TU.

##### TACF arm

The NTEP HCW will screen the index patient for TB via telephone calls at 6 and 12 months post-treatment completion; the index patient will also be asked about TB symptoms among HHCs. If TB is suspected among any HH members, we will ask them to visit the TU to provide a spot sputum specimen for microbiological testing at the TU.

##### TB events

A two-part TB event questionnaire will be administered to individuals identified with new or recurrent TB during or between study time points. Part one of the questionnaire will be administered shortly after diagnosis and will include self-reported duration of TB symptoms. Part two will be administered at month 18 (“mop-up”) or at the end of that individual’s TB treatment, whichever is earlier.

##### “Mop-up” campaign

To determine total number of TB diagnoses across HHs in both arms, three strategies will be undertaken. First, all index patient names will be periodically cross-matched with the NTEP registry. Second, on all calls and at all home visits, participants will be asked if there were any TB diagnoses in the period since the last call/visit and when reported will be verified with the NTEP. Third, all HHs in both arms will have an 18-month home visit to screen all HH members for active TB and to survey them for history of TB. We will use the “mop-up” campaign data combined with the registry match data to account for all TB cases occurring during the study period. This will allow for more accurate comparisons between arms and to calculate proportion of overall recurrent and HH TB cases that were captured by our ACF strategies during the study period. In addition, we will compare timing of TB diagnoses between arms, as well as timing of TB detected by our ACF strategies vs. otherwise.

##### Exposures of interest

- *Biomass fuel:* HH biomass fuel use will be assessed using self-reported questionnaires previously validated in our setting [60, 61].
- *Smoking:* We will measure exposure to tobacco smoke, nicotine dependence based on the Fagerström Test for Nicotine Dependence, and heaviness of smoking among participants at entry [62].
- *Alcohol:* We will measure alcohol use in adult (>= 18 years) participants at entry using the Alcohol Use Disorders Identification Test (AUDIT), which has been validated in India [63].
- *Point-of-care HbA1c*: To identify diabetes, defined as HbA1c ≥6.5%, we will measure HbA1c among participants at entry using advanced fluorescent immunoassay technology (Standard F200, SD Biosensor, Republic of Korea). This point-of-care HbA1c test has been validated and approved by Indian regulatory authorities.
- *HH socio-economic status:* Participants will describe their type of residence, the number of occupants, type of employment of employed individuals, and monthly HH income.

Visited HHs in both arms will be geocoded using android devises via Redcap during home visits. HHs not visited in the TACF arm will be geocoded during the “mop-up” visit. The residential location will be further characterized by collecting HH-level data such as: predominant housing type, sanitation and infrastructure, transportation access, and distance to closest TU.

#### Objective 2: data collection

We will use the RE-AIM framework to organize our assessment of implementation outcomes. We will focus on the following domains from RE-AIM, with additional attention to intervention acceptability [54].

##### (R) Reach

We will identify sub-populations that are best reached by the intervention and sub-populations who may benefit the most from the intervention. We will assess reach among enrolled participants in terms of actually fully receiving either ACF strategy. This will be determined through intervention logs and tracking tools.

##### (E) Effectiveness

The effectiveness of the two ACF strategies will be measured through our primary and secondary outcomes.

##### (A) Adoption and acceptability

We will assess adoption and acceptability using a mixed-methods approach. All interviews with index patients, HHCs, and HCWs will be conducted post final study visit (post 12-month endpoint) to ensure there is no contamination and to use 12-month outcomes for selecting participants. The interviews will be conducted by trained behavioral scientists fluent in local languages (Marathi and Hindi). The open-ended segment of interviews will be audio-recorded (with participant’s consent) and will be transcribed and translated into English as necessary. Our exploration of adoption and acceptability will be guided by a theory-based perspective using the Normalization Process Theory (NPT) [64–66]. Acceptability will be guided by the work of Sekhon et al. [67]. We will also explore perceptions of and experiences with the ACF strategies including (a) participant’s perceptions of alignment of services with needs, (b) confidentiality or other concerns with home visits, and (c) perceptions of the quality of services provided. The first component of the interview will include open-ended questions with probes, while the second half of the session will include structured survey questions on these domains.

##### (I) Implementation

Implementation will be assessed based on fidelity to the predetermined components, steps, and procedures of each ACF strategy quantitatively using intervention logbooks and tracking tools.

##### (M) Maintenance

The potential for maintenance will be assessed through the cost and cost-effectiveness analyses to be conducted in objective 3.

#### Objective 3: data collection

**Fig. 2:**
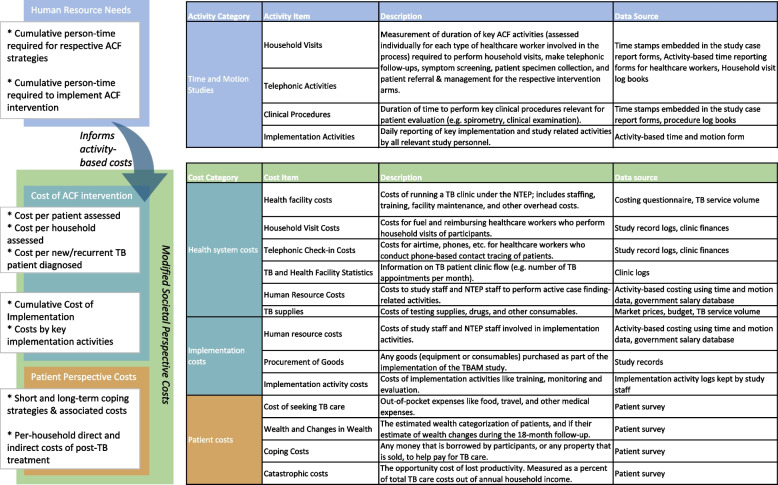
Summary of major cost items and data sources

We will conduct a suite of economic evaluations and a modeling study based on empiric data collected during the trial. Figure 2 summarizes all economic cost data to be collected in the trial and data sources.

##### Time and motion studies

ACF operations will be assessed based on the WHO’s costing guidelines for TB interventions using an activity-based costing framework [68]. Cost data will be collated based on activities performed directly versus indirectly to the study participants and by resource type. Data on activity-based resource use will be assessed based on time and motion studies [69, 70]. These data will then be used to apportion program implementation costs, overhead costs, and infrastructural costs per patient screened and per patient diagnosed with TB. All resource use and cost data will be collected based on standardized cost data collection instruments [71, 72].

##### Health system and implementation costs

Costs will primarily be considered from the perspective of the NTEP as the party responsible for financing TB diagnosis and treatment in India. We will assess costs of implementing the intervention using a standardized, three-phase framework developed by our team [73]. For each implementation phase, we will collate resource-use and cost data for each pre-defined activity through periodic review of study activity reports/logs, financial documents, and discussions with study staff. Human resource time commitments will also be tracked for each study activity using a combination of study staff/HCW time sheets, activity logs, and structured interviews.

##### Patient costs

The WHO’s Tuberculosis Patient Cost Survey was designed as a cross-sectional survey and does not incorporate post-treatment costs [74]. TB Aftermath provides an opportunity to overcome these limitations by longitudinally surveying patients on costs experienced during and after TB treatment. Our modified survey also measures costs associated with the patient’s initial TB episode, any recurrence, and any new TB episodes among their HHCs. For index patients, pre-treatment and treatment phase costs will be assessed at study enrolment and post-treatment costs will be assessed at the months 6 and 18 (“mop-up”) time points. Specifically, we will measure wealth, changes in income and employment status, direct out-of-pocket costs of TB care, and borrowing and selling of assets to cope with illness during and after treatment.

#### Objective 4: data collection

We will measure lung function and respiratory health status at enrolment and month 18 (“mop-up”) in all participants and semi-annually in the home-based arm only. Lung function will be assessed by point-of-care pre-bronchodilator spirometry performed by trained study staff according to American Thoracic Society and European Respiratory Society guidelines [75]. We will collect key lung function indices such as forced expiratory volume in the 1st second (FEV1), forced vital capacity (FVC), and peak expiratory flow rate (PEF) at enrolment and follow-up. Additionally, we will measure respiratory health status and quality of life from the patient perspective using standardized questionnaires such as the Saint Georges Respiratory Questionnaire and the COPD Assessment Test [76–78].

### Plans to promote participant retention and complete follow-up {18b}

At entry, study staff will record the phone number of one HHC in case the index patient is not reachable or changes their phone number during follow-up. For the HACF arm, visits will be scheduled at a time that is convenient for both the participant and the NTEP HCW to minimize missed follow-up visits. For the TACF arm, we will make a minimum of three attempts (at different times of day) to contact patients telephonically at each interval. The study team will meet biweekly to discuss field-level challenges, and strategies for improving retention.

### Data management {19}

This study will use electronic case report forms (eCRFs). If any data is recorded directly on the eCRF (i.e., no prior written or electronic record of data), that eCRF will be considered the source document. eCRFs will be designed using RedCap (v12.0.16) with an offline data entry option using an android mobile app. Other source documentation may include, but is not limited to, photocopies of hospital/clinic medical records, progress notes, laboratory results, and radiology results. We will use the built-in RedCap audit trail log for documenting all changes to the database during the study. All study staff will follow instructions specified in the data quality management SOP, which requires running periodic queries to check data quality.

### Confidentiality {27}

All mobile devices will be encrypted. The following procedures will be followed to protect the confidentiality of data collected and stored:

- Only authorized persons will be granted access
- Only authorized persons may enter and view study data
- Passwords and system IDs will not be shared
- Physical security of the workstations/files will be maintained
- Adequate back-up plan is in effect
- Staff trained on data entry system and importance of security procedures
- Workstations with databases will not be left unattended

For the qualitative data collection, audio recordings of interviews will be transcribed and audio files will be destroyed once data analysis is complete. Identifying information will be removed from transcripts and unique identification number assigned at entry will be used. Upon completion of the study, data will be stored in an on-site data storage facility for at least five years after the last participant’s follow-up is completed.

### Plans for collection, laboratory evaluation, and storage of biological specimens for genetic or molecular analysis in this trial/future use {33}

N/A. Biological specimens will not be stored.

## Statistical methods

### Statistical methods for primary and secondary outcomes {20a}

The primary analysis will be intention-to-treat, which will include all enrolled participants who have completed TB treatment and their household contacts. The primary endpoint is rate of TB disease detected by study arm, which includes recurrent TB among index cases and new TB among HHCs including children. To assess non-inferiority of TACF, compared to HACF, we will estimate a 95% confidence interval around the difference between the observed TB cases diagnosed between arms and establish non-inferiority if the upper bound of this estimate is less than 1.7 per 100 person-years, the non-inferiority margin. We will analyze impact of key risk factors for TB disease (tobacco smoking, biomass fuel use, alcohol use, diabetes, respiratory impairment, low socioeconomic status, catastrophic costs) on the development of recurrent TB and new TB among HHCs. We will use mixed-effects Poisson regression with random effects for HH and fixed effects for individual level variables to measure the association of key risk factors with recurrent and new TB disease in the HH. For the secondary outcome, a logistic regression model with mixed effects, to account for HH level clustering, will be used to determine if HACF is independently associated with TPT initiation among children < 6 years of age.

### Interim analyses {21b}

Intervention allocation will remain concealed for any interim analyses conducted.

### Methods for additional analyses (e.g., subgroup analyses) {20b}

#### Objective 1: additional analyses

Subgroup analyses will be conducted to further explore our primary and secondary outcomes. For example, as TPT is now recommended for all HHCs in India [8], we will expand upon our secondary outcome to assess proportion of HHCs (regardless of age) who initiate TPT after ruling out TB disease, by study arm.

#### Objective 2: analyses

##### Mixed-methods approach and thematic analysis

Our approach to the qualitative data will involve thematic analysis and employ both inductive and deductive coding techniques [79]. The software MAXQDA software will be used to code the textual data. We will first develop an *a priori* code book that reflects key analytic concepts and additional codes may be added to document emerging themes of interest. Textual analysis will proceed by first exploring broad patterns and experiences of study participants and then assessing possible similarities and differences in experiences between sub-groups. Quantitative data from the structured close-ended questions will be analyzed using descriptive statistics, characterizing the median and distribution of scores by sub-groups. We will integrate findings from qualitative and quantitative analyses to gain an in-depth understanding of implementation outcomes with a focus on adoption and acceptability.

##### Reach

We will use descriptive statistics to determine the proportions of socio-demographic groups that (a) receive the intervention as planned, and (b) are diagnosed with TB. We will use mixed effects logistic regression or log-binomial regression, including clinic as a mixed effect, to assess for associations between service receipt (e.g., home visit or telephone call) and the factors of interest. We will use mixed effects logistic regression to assess associations with recurrent TB diagnosis among the index patient and, separately, TB diagnosis among HHCs.

##### Implementation

We will quantitatively assess indicators of implementation fidelity including (a) whether telephone contact numbers correct and available, (b) proportion of HHs respond to phone calls, and (c) proportion of HHs located and visited. We will use a goal of 90% delivery of components considered core and able to be controlled by the health system. If a lower level of delivery is achieved in any of these components, we will explore potential reasons in in the open-ended interview segment.

### Objective 3 analyses

#### Model for recurrence and care-seeking

Our model will simulate HHs of former patients with TB, basing HH size and demographics on participating trial HHs. The probabilities of TB recurrence and of new cases among HHCs at each time step will be modeled as a parametric (inverse Gaussian) function of time since index patient treatment (with a second peak of TB risk for contacts following any recurrent TB in the HH), and care seeking probability will be a function of an individual’s time with TB symptoms. Starting with prior estimates informed by existing literature, parameters reflecting cumulative TB risk, distribution of time to TB onset, and time to diagnosis once symptomatic will be calibrated to trial data, using a Bayesian process to select estimates that most closely and consistently replicate trial results while remaining consistent with known TB epidemiology. Data from the trial that will be used for calibration include the total numbers of (a) recurrent TB cases and (b) TB among HHCs diagnosed during each 6-month follow-up interval, (c) the numbers detected at each screening visit for each arm (corresponding to individuals who are symptomatic but undiagnosed at 6 months or 12 months in the model), and the duration of symptoms reported (d) by those cases who are detected through ACF and (e) by those who were found through routine care (as reported at each study visit or detected through NTEP registry review).

Additional components of the model—including the treatment of recurrent and household TB once detected, the outcomes of undiagnosed TB, and the impact of TPT if delivered—will be based on our experience with Markov modeling of other TB-related interventions [80–84]. Among children, we will model the TPT coverage reported before the trial, and for ACF interventions, we will include the additional TPT coverage delivered during the trial, with resulting reduction in subsequent TB risks. The model will be extended over a lifetime time horizon, for purposes of comparing over simulated lifetimes the number of TB cases averted, deaths averted, years of life lost averted, and years of life with disability averted by different ACF strategies.

#### Modeling population impact of ACF interventions

We will use a population-level model to estimate the impact of each ACF strategy on TB incidence. Our model will integrate (a) estimated reductions in time spent with infectious TB in a Markov model of HACF and TACF, (b) the estimated proportion of TB in India occurring among former TB patients [21, 85], and (c) the relative number of TB transmission events generated within versus outside of an index patient's household in order to estimate resulting reductions in force of infection [86, 87]. Our dynamic transmission model will be calibrated to India’s current TB epidemic, similar to others we have previously developed [84, 88], to estimate the reduction in TB incidence that could be achieved through 10 years of widespread implementation of TACF or HACF.

#### Exploring variations on trial interventions

We will also use our Markov model of HACF and TACF described above to simulate alternative schedules and more targeted applications for those interventions. Simulated schedules may include shifting the screening visits to earlier (e.g., 3 and 6 months) or later (e.g., adding a third visit at 18 months). We will use study data on the distribution of risk factors and the associated relative risks for recurrent and household TB in the Indian population to simulate strategies which target ACF to high-risk patients.

#### Cost analyses

Intervention costs will be categorically assessed to include costs of the implementation stages of the respective intervention [73]. All costs will be assessed in units of current-year US dollars, with capital assets, up-front costs (e.g., implementation costs), and other fixed costs annuitized using a 3% annual discount rate (varied between 0 and 7% in sensitivity analyses), and each item’s relevant expected useful life years. The primary outcome for health systems cost will be average cumulative per-household cost of each ACF strategy. This estimate will include combined costs of the process of intervention implementation and delivery. We will also describe major cost drivers for each strategy and conduct sensitivity and scenario analyses around the major factors influencing the primary cost estimate. For patient and household-level factors, we will use multivariable regression techniques to develop cost functions that describe the relationships between patient and household-level variables (e.g., gender, distance from clinic, duration of smoking) and patient costs under each intervention condition. For patient costs, the primary outcome will be assessed as total economic cost per household with index TB patient, categorically assessed by the type of TB episode(s) experienced (initial, recurrent, and/or new). We will also calculate the percentage of TB illness related economic costs out of the household income to assess whether the index patient’s household experienced catastrophic costs (defined as TB related patient costs exceeding 20% of the household income) [74, 89].

#### Cost-effectiveness

Both incremental costs and effectiveness will be estimated relative to no intervention primarily and for HACF relative to TACF secondarily. Cost-effectiveness will first be determined based on empiric cost estimates of the respective strategies and the primary outcome assessed in objective 1. Therefore, the primary cost-effectiveness outcome will be assessed as incremental cost (whichever strategy has higher effectiveness) per additional TB episode (new or recurrent) identified through the intervention.

The secondary measure of cost-effectiveness will be the incremental cost per incremental DALY averted. DALYs averted will be based on estimated numbers of TB cases prevented, improved treatment outcomes, and deaths averted using disability weights from the 2017 Global Burden of Disease study [90, 91]. Cost-effectiveness will be evaluated from the health system perspective over a lifetime time horizon, with future costs and effectiveness discounted at 3% annually. The incremental cost-effectiveness ratio will be calculated comparing HACF and TACF versus the standard of care (no ACF), comparing different ACF schedules for ACF strategies which appeared cost-effective, and considering the cost-effectiveness of targeted ACF (based on identifiable risk factors in the index patient) for strategies that do not appear cost-effective when delivered to all former TB patients. Accepted methodological guidelines will be followed for the conduct, and reporting of the cost-effectiveness analysis [92–94].

#### Sensitivity analysis

We will perform extensive one-way and multi-way sensitivity analyses to evaluate the key drivers of impact, cost, and cost-effectiveness in the Indian context and will perform probabilistic uncertainty analyses to quantify the level of uncertainty in our cost-effectiveness estimates.

### Objective 4: analyses

The degree of lung impairment in TB cases will be assessed by comparing with apparently healthy controls with no evidence of TB disease. Lung function parameters will be z-score standardized using Global Lung Initiative (GLI) reference equations [95]. Impaired lung function parameters will be defined as having an observed parameter value <5th percentile of the expected parameter distribution for a given age, sex, and height using the GLI equations. We will measure the association between impaired FEV1, FVC and FEV1/FVC ratio, and TB recurrence using Poisson regression. Our secondary analysis will measure the association between change in respiratory questionnaire scores and lung function parameters during follow-up, and subsequent TB recurrence using mixed-effects Poisson regression.

### Methods in analysis to handle protocol non-adherence and any statistical methods to handle missing data {20c}

All deviations from the protocol will be addressed in the study participant’s source documents.

### Plans to give access to the full protocol, participant level-data and statistical code {31c}

The final de-identified trial dataset and statistical code will be made available by the corresponding author on reasonable request.

### Oversight and monitoring

#### Composition of the coordinating center and trial steering committee {5d}

Dr. D.Y. Patil Medical College, Hospital and Research Centre, Dr. D.Y. Patil Vidyapeeth, located in the Pimpri-Chinchwad Municipal Corporation area of Pune district, India, serves as the coordinating center running day-to-day trial operations with organizational and scientific support from JHU located in Baltimore, Maryland. JHU also serves as the coordinating center for regulatory tracking specifically. A core group of study coordinators and investigators meets at least biweekly. The Maharashtra State TB Office oversees the trial, with formal meetings held at least annually.

#### Composition of the data monitoring committee, its role and reporting structure {21a}

N/A. A data monitoring committee was not included for TB Aftermath, as the trial is a hybrid effectiveness-implementation trial with minimal risk to participants, and the activities included are an extension of usual care by HCWs.

#### Adverse event reporting and harms {22}

N/A. There is no more than minimal risk associated with study procedures for any of the objectives and thus, adverse events are not anticipated.

#### Frequency and plans for auditing trial conduct {23}

During the study period, an internal monitor will review trial conduct on an annual basis. The internal monitor is an employee of Johns Hopkins India who is not otherwise involved in the conduct of the trial.

#### Plans for communicating important protocol amendments to relevant parties (e.g., trial participants, ethical committees) {25}

If a protocol deviation meets the following criteria, it will be reported to the IRB and the sponsor: (1) results in a significant added risk to the study participant occurs when the participant or investigator has failed to adhere to protocol requirements impacting on enrolment eligibility, safety surveillance, and endpoint outcomes and (2) when there is non-adherence to Good Clinical Practice standards. Examples of reportable protocol deviations include eligibility violations, informed consent violations, and confidentiality violations. Furthermore, protocol deviations that violate eligibility criteria, informed consent processes, or participant confidentiality must be reported immediately to the local IRB, per their requirements. All other protocol deviations (those that do not meet the criteria outlined above) will be maintained in a log that is submitted to the IRB with annual progress reports.

#### Dissemination plans {31a}

Study results will be disseminated between years 3 and 5 of the study. Meetings will be held with NTEP officials to share results and discuss policy implications. Materials and/or publications generated under the project will be disseminated in accordance with the participating institution and NIH policies.

## Discussion

Implementing ACF is a recognized need for TB control in high incidence settings. However, the comparative effectiveness of ACF strategies among treated TB patients has not been assessed despite the high risk of recurrence among this populations and the high rate of TB among their HHCs. Using a hybrid effectiveness-implementation approach and collaborating with the Indian NTEP will allow for rapid scale-up of the strategy(ies) deemed to be successful. To strengthen generalizability of results, our study is embedded into the program and utilizes existing NTEP HCWs. Additionally, measuring the costs and cost-effectiveness of these strategies and assessing risk factors for recurrence will provide insight into prioritization of subpopulations when ACF for treated TB patients is implemented. This novel trial will guide India’s scale-up of post-treatment ACF and provide an evidence base for designing strategies to detect recurrent and new TB in other high burden settings.

## Trial status

This paper corresponds with protocol version 1.3, January 28, 2022. Recruitment began on January 29, 2021, and the approximate completion date for recruitment is February 1, 2023.

### Supplementary Information

**Additional file 1.** Original Funding Documentation.

**Additional file 2.** JHU Ethical Approval.

**Additional file 3.** DPU Ethical Approval.

## Data Availability

The final de-identified trial dataset will be made available by the corresponding author on reasonable request.

## Abbreviations

ACF: Active case finding
AUDIT: Alcohol Use Disorder Identification Test
DALY: Disability-adjusted life years
DOT: Directly observed therapy
eCRF: Electronic case report form
HCW: Health care worker
HH: Household
HACF: Home-based active case finding
HHC: Household contacts
HIV: Human immunodeficiency virus
IRB: Institutional Review Board
JHU: Johns Hopkins University
NIAID: National Institute of Allergy and Infectious Diseases
NIH: National Institutes of Health
NTP: National Tuberculosis Program
NTEP: National Tuberculosis Elimination Program
PI: Principal investigator
RE-AIM: Reach, Effectiveness, Adoption & Acceptability, Implementation, Maintenance
STLS: Senior TB laboratory supervisor (STLS)
STS: Senior TB treatment supervisor
TACF: Telephonic active case finding
TB: Tuberculosis
TPT: Tuberculosis preventive treatment
TU: Tuberculosis unit
WHO: World Health Organization
