# Correction: Tuberculosis (TB) Aftermath: study protocol for a hybrid type I effectiveness-implementation non-inferiority randomized trial in India comparing two active case finding (ACF) strategies among individuals treated for TB and their household contacts

**DOI:** 10.1186/s13063-024-07984-3

**Published:** 2024-03-11

**Authors:** Samyra R. Cox, Abhay Kadam, Sachin Atre, Akshay N. Gupte, Hojoon Sohn, Nikhil Gupte, Trupti Sawant, Vishal Mhadeshwar, Ryan Thompson, Emily Kendall, Christopher Hofmann, Nishi Suryavanshi, Deanna Kerrigan, Srikanth Tripathy, Arjunlal Kakrani, Madhusudan S. Barthwal, Vidya Mave, Jonathan E. Golub, the TB Aftermath study team

**Affiliations:** 1 Johns Hopkins Bloomberg School of Public Health, 615 N Wolfe St, Baltimore, MD 21205 USA; 2 Johns Hopkins School of Medicine, 600 N Wolfe St, Baltimore, MD 21287 USA; 3 Johns Hopkins India, G-4 & G-5, PHOENIX Building, OPP. to Residency Club, Pune, Maharashtra 411001 India; 4 Dr. D.Y. Patil Medical College, Hospital and Research Centre, Dr. D.Y. Patil Vidyapeeth, Sant Tukaram Nagar, Pimpri Colony, Pimpri-Chinchwad, Maharashtra 411018 India; 5 Seoul National University College of Medicine, 103 Daehak-Ro, Jongno-Gu, Seoul, 03080 Republic of Korea; 6 George Washington University, 2121 I St NW, Washington, D.C, 20052 USA

**Correction: Trials 23, 635 (2022)**

**https://doi.org/10.1186/s13063-022-06503-6**

Following publication of the original article [1], we have been notified that sputum collection has been done at the TU instead of at home, as originally stated in the paper. This change was made due to logistical constraints. The Interventions and Data collection and management sections have been modified to reflect this.

In the Sample size section, second paragraph, the phrase “*Assuming a rate of 12 TB cases per 100 person-years (…) we are powered at 80% to determine that the TACF arm is non- inferior to the HACF arm with a non-inferiority interval of 3 per 100 person-years and a sample size of 1076 index patients and two HHCs per index patient (n* = *2152)*” was also modified into “*Assuming a rate of 12 TB cases per 100 person-years (…) we are powered at 90% to determine that the TACF arm is non- inferior to the HACF arm with a non-inferiority interval of 1.7 per 100 person-years and a sample size of 1076 index patients and two HHCs per index patient (n* = *2152)*” (changes marked in bold).

3 was also changed into 1.7 into the Statistical Methods section (“the upper bound of this estimate is less than 1.7 per 100 person-years”). 3 per 100 person-years applied to recurrent cases only. The non-inferiority margin for the primary outcome which includes both new and recurrent cases is 1.7 per 100 person-years.

The author contribution section was also modified to include the statement: “SA and TS resigned from the study and were not involved in the discussion related to the manuscript correction.”

The original article has been corrected.
