# Supplementary material for: Tuberculosis (TB) Aftermath: study protocol for a hybrid type I effectiveness-implementation non-inferiority randomized trial in India comparing two active case finding (ACF) strategies among individuals treated for TB and their household contacts
Source: Trials. 2022 Aug 5;23:635. doi: 10.1186/s13063-022-06503-6 (PMC9354295; doi:10.1186/s13063-022-06503-6)
Supplement: Supplementary file 1 — Additional file 1. Original Funding Documentation. [file 13063_2022_6503_MOESM1_ESM.pdf]

RESEARCH
Department of Health and Human Services
National Institutes of Health

Federal Award Date: 05/26/2020

NATIONAL INSTITUTE OF ALLERGY AND INFECTIOUS DISEASES

Grant Number: 1R01AI143748-01A1
FAIN: R01AI143748

Principal Investigator(s):
JONATHAN E GOLUB (contact), PHD
Vidya Mave, MD

Project Title: TB Aftermath

Ms. Alston-Rodgers, Katrina
Sr. Grants Associate
733 N Broadway, Suite 117
Baltimore, MD 212051832

Award e-mailed to: eawards@jhu.edu

Period Of Performance:
Budget Period: 06/01/2020 – 05/31/2021
Project Period: 06/01/2020 – 05/31/2025

Dear Business Official:

The National Institutes of Health hereby awards a grant in the amount of $695,483 (see “Award Calculation” in Section I and “Terms and Conditions” in Section III) to JOHNS HOPKINS UNIVERSITY in support of the above referenced project. This award is pursuant to the authority of 42 USC 241 42 CFR 52 and is subject to the requirements of this statute and regulation and of other referenced, incorporated or attached terms and conditions.

Acceptance of this award including the “Terms and Conditions” is acknowledged by the grantee when funds are drawn down or otherwise obtained from the grant payment system.

Each publication, press release, or other document about research supported by an NIH award must include an acknowledgment of NIH award support and a disclaimer such as “Research reported in this publication was supported by the National Institute Of Allergy And Infectious Diseases of the National Institutes of Health under Award Number R01AI143748. The content is solely the responsibility of the authors and does not necessarily represent the official views of the National Institutes of Health.” Prior to issuing a press release concerning the outcome of this research, please notify the NIH awarding IC in advance to allow for coordination.

Award recipients must promote objectivity in research by establishing standards that provide a reasonable expectation that the design, conduct and reporting of research funded under NIH awards will be free from bias resulting from an Investigator’s Financial Conflict of Interest (FCOI), in accordance with the 2011 revised regulation at 42 CFR Part 50 Subpart F. The Institution shall submit all FCOI reports to the NIH through the eRA Commons FCOI Module. The regulation does not apply to Phase I Small Business Innovative Research (SBIR) and Small Business Technology Transfer (STTR) awards. Consult the NIH website http://grants.nih.gov/grants/policy/coi/ for a link to the regulation and additional important information.

If you have any questions about this award, please contact the individual(s) referenced in Section IV.

Sincerely yours,

Gregory P. Smith
Grants Management Officer
NATIONAL INSTITUTE OF ALLERGY AND INFECTIOUS DISEASES

Additional information follows

NIH NGA R | Version: 56 - 12/26/2018 2:22:00 PM| Generated on: 5/26/2020 12:03:38 AM
