# Supplementary material for: Tuberculosis (TB) Aftermath: study protocol for a hybrid type I effectiveness-implementation non-inferiority randomized trial in India comparing two active case finding (ACF) strategies among individuals treated for TB and their household contacts
Source: Trials. 2022 Aug 5;23:635. doi: 10.1186/s13063-022-06503-6 (PMC9354295; doi:10.1186/s13063-022-06503-6)
Supplement: Supplementary file 2 — Additional file 2. JHU Ethical Approval. [file 13063_2022_6503_MOESM2_ESM.pdf]

Office of Human Subjects Research
Institutional Review Boards

1620 McElderry Street, Reed Hall, Suite B-130
Baltimore, Maryland 21205-1911
410-955-3008
410-955-4367 Fax
e-mail: jhmeirb@jhmi.edu

Date: June 8, 2020

APPLICATION APPROVAL

**Review Type:**	Expedited
**Principal Investigator:**	Jonathan Golub
**Number:**	IRB00247239
**Title:**	Tuberculosis (TB) Aftermath
**Committee Chair:**	Susan Bassett
**IRB Committee:**	IRB-X

Date of Approval: June 5, 2020

Date of review of Administrative Changes: June 5, 2020

Date of Expiration: May 28, 2021

The JHM IRB approved the above-referenced Application.

The JHM IRB approved this research, however due to the human subject research restrictions imposed by the Institution, you may not enroll new participants in the study until the restrictions are lifted. Before proceeding with this study, please consider the restrictions and recommendations made by local authorities and the local IRB regarding continuation of research during the COVID-19 pandemic. Please review information at the following link for guidance on international studies during the pandemic:
https://hub.jhu.edu/novel-coronavirus-information/research-preparedness/research-preparedness-human-subjects/human-subject-research-international-supplemental-guidance/

To keep the JHM IRB application current we are assigning an Expiration Date as noted above. Prior to the expiration date, you will receive an email notification indicating that some action is required. If the Board has determined that a Continuing Review or Progress Report is required, you will need to submit Continuing Review or Progress Report prior to the expiration date. If the Board has determined that No Progress Report is required, you may run the administrative extend approval function.

IRB review included the following:

Clinical trials must be registered with a clinical trials registry that is electronically searchable and accessible to the public at no charge (i.e.: http://www.clinicaltrials.gov) as required by the September, 2007 FDA Amendments Act (affecting new and ongoing trials as of January 25, 2008). If this is a commercially sponsored trial, the Hopkins PI should consult with the commercial sponsor to assure that posting of the trial is in accord with terms of the study contract.

The enrollment of the non-English speaking participants. If you are using a short form consent, follow the JHM IRB Guidance on Obtaining and Documenting Informed Consent of Subjects Who Do Not Speak English.

Progress Report Required:

The Board determined that this research meets the criteria for submission of a Progress Report as an alternative to a Continuing Review Application. The Progress Report must be submitted using a Further Study Action and selecting progress report at least 6 weeks prior to the expiration date. Please note, the Progress Report **must** be submitted prior to the expiration date shown on this notice. If the Progress Report is not submitted prior to the expiration date all activity must stop. Before any research activity can resume, you must submit the progress report.

45CFR46.404 and/or 21 CFR 50.51: This study has been approved for the inclusion of children as 'research not involving greater than minimal risk'. The permission of one parent is required.

Assent is waived for some children.

Assent via the child's signature is required for children over 9 years of age.

Collection of blood samples by finger stick, heel stick, ear stick, or venipuncture from healthy, nonpregnant adults who weigh at least 110 pounds. For these subjects, the amounts drawn may not exceed 550 ml in an 8 week period and collection may not occur more frequently than 2 times per week; or from other adults and children, considering the age, weight, and health of the subjects, the collection procedure, the amount of blood to be collected, and the frequency with which it will be collected. For these subjects, the amount drawn may not exceed the lesser of 50 ml or 3 ml per kg in an 8 week period and collection may not occur more frequently than 2 times per week.

Prospective collection of biological specimens for research purposes by noninvasive means.

Research on individual or group characteristics or behavior (including, but not limited to, research on perception, cognition, motivation, identity, language, communication, cultural beliefs or practices, and social behavior) or research employing survey, interview, oral history, focus group, program evaluation, human factors evaluation, or quality assurance methodologies.

Changes in Research: All proposed changes to the research must be submitted using a Change in Research application. The changes must be approved by the JHM IRB prior to implementation, with the following exception: changes made to eliminate apparent immediate hazards to participants may be made immediately, and promptly reported to the JHM IRB.

Unanticipated Problems: All unanticipated problems must be submitted using a Protocol Event Report.

If this research has a commercial sponsor, the research may not start until the sponsor and JHU have signed a contract.

Study documents:

Written Consent:

Only consent forms with a valid approval stamp may be presented to participants. All consent forms signed by subjects enrolled in the study should be retained on file. The Office of Human Subjects Research conducts periodic compliance monitoring of protocol records, and consent documentation is part of such monitoring.

Written Assent:
Study will not be using a Hopkins Assent document.docx

Additional Supplemental Study Documents:
Appendix 1.1_Study Prep_HCW Interview Guide_V1.1.docx
Ethics Approval_TB Aftermath.pdf
Appendix 1.2_Aim 1_Exposures of Interest Questionnaires_v1.1.pdf
Appendix 1.3_Aim 2_Interview Guides_V1.1.docx
TB_Aftermath-Approval_IT_Data_Trust.pdf

Protocol:
TB Aftermath Protocol_ V 1.1 _Clean_April 23 2020_FINAL.docx

Johns Hopkins Study Team Members:

Samyra Cox, Hojoon Sohn, Emily Kendall, Nikhil Gupte, Vidya Mave, Akshay Gupte, David Dowdy, Christopher Hoffmann, Matthew Riesner

The Johns Hopkins Institutions operate under multiple Federal-Wide Assurances: The Johns Hopkins University School of Medicine - FWA00005752, Johns Hopkins Health System and Johns Hopkins Hospital - FWA00006087
