# Supplementary material for: Tuberculosis (TB) Aftermath: study protocol for a hybrid type I effectiveness-implementation non-inferiority randomized trial in India comparing two active case finding (ACF) strategies among individuals treated for TB and their household contacts
Source: Trials. 2022 Aug 5;23:635. doi: 10.1186/s13063-022-06503-6 (PMC9354295; doi:10.1186/s13063-022-06503-6)
Supplement: Supplementary file 3 — Additional file 3. DPU Ethical Approval. [file 13063_2022_6503_MOESM3_ESM.pdf]

DPU

ETHICS COMMITTEE
Dr.D.Y.Patil VIDYAPEETH, PUNE

(Re-Reg.No.ECR/361/Inst/MH/2013/RR-16 : issued under rule 122DD of drugs & cosmetics rules1945)

Dr.D.L.Ingole
Chairman

Dr (Brig) Daniel Saldanha
Member Secretary

Ref.No.DYPV/EC /382 / 2019

Date: 25 November 2019

CERTIFICATE

To,
Dr.M.S.Barthwal
Department of Respiratory medicine,
Dr.D.Y.Patil Medical college Hospital & research centre Pimpri, Pune-18.

Subject: - Ethics Committee Approval for the Study

Dear Dr.M.S.Barthwal,

The Ethics Committee-Dr.D.Y.Patil Vidyapeeth has reviewed and discussed your application to conduct the study entitled **"TB Aftermath."**

The following documents were reviewed:

1. Protocol version 1.0
2. Consent English, Hindi, Marathi version 1.0
3. C.V of P.I

The following members of the ethics committee were present at the meeting held on **23/11/2019** 11:00 am at the Board Room, Dr. D.Y. Patil Vidyapeeth, Pimpri Pune - 411018.

Ethics Committee Dr.D.Y.Patil Vidyapeeth, Pune
Pimpri, PUNE - 18

ETHICS COMMITTEE
Dr.D.Y.Patil VIDYAPEETH, PUNE

DPU

(Re-Reg.No.ECR/361/Inst/MH/2013/RR-16 : issued under rule 122DD of drugs & cosmetics rules1945)

Dr.D.L.Ingole
Chairman

Dr (Brig) Daniel Saldanha
Member Secretary

Ref.No.DYPV/EC / 382 / 2019

Date: 25 November 2019

Sr. No.	Name of Members	Designation with Address	Position in EC
1	Dr. D.L. Ingole	Retired :<br>"Dattaprasad" Bhukrupa Housing, Sector 27 J, NigdiPradhikaran, Pune-411044.	Chairperson<br>(Clinician)
2	Dr.Ravinder Singh	Scientiest "C", Division of NCD 19 CMR, Ansari Nagar, New Delhi.	Member<br>(Scientist-C)
3	Dr.M.V.Khadilkar	Technical Director –Premium Serum and Vaccines Pvt.Ltd., Narayangaon	Member<br>(Scientist)
4	Dr.R.K.Nanda	Professor, Dr. D.Y. Patil Pharmacology, Pimpri,<br>Pune-18.	Member<br>Basic Scientist Pharmacology
5	Adv.Dr.Ruby Chhatwal	Sai-Ambience, 'F' Building, Flat No. 502, Pimple-Saudagar, Pune-411027.	Member<br>(Social Scientist)
6	Dr.Neelu Nawani	Professor, Dr.D.Y.Patil Biotechnology & Bioinformatics Institute, Tathawade, Pune-33.	Member<br>(Basic Scientist)
7	Dr.(Mrs) Shubhangi Abhay Kanitkar	Professor, Dept. of Medicine, Dr.D.Y.Patil Medical College & Hospital, Pimpri, Pune-18.	Member<br>(Clinician)
8	Dr Suprakash Chaudhury	Professor, Dept. of Psychiatry, Dr.D.Y.Patil Medical College & Hospital Research Centre, Pimpri, Pune-411018.	Member<br>(Clinician)
9	Mrs.Beena Gokhale	Nandadeep Society, Durgeswar Path Road, Opp. Durgeswar Mandir, Nigadi Pune-18.	Lay Person
10	Adv.Dr.Ruby Chhatwal	Sai-Ambience, 'F' Building, Flat No. 502, Pimple-Saudagar, Pune-411027.	Member<br>(Social Scientist)
11	Dr. Amit Jagtap	Professor.Prosthodontics, Dr. D. Y. Patil Dental College & Hospital, Pimpri, Pune	Member<br>(Clinician)
12	Dr.(Brig.) Daniel Saldanha	Prof. & Head, Dept. of Psychiatry, Dr.D.Y.Patil Medical College & Hospital Research Centre, Pimpri, Pune-411018.	Member Secretary

Ethics Committee Dr.D.Y.Patil Vidyapeeth, Pune
Pimpri, PUNE - 18

DPU
ETHICS COMMITTEE
Dr.D.Y.Patil VIDYAPEETH, PUNE

(Re-Reg.No.ECR/361/Inst/MH/2013/RR-16 : issued under rule 122DD of drugs & cosmetics rules1945)

Dr.D.L.Ingole
Chairman

Dr (Brig) Daniel Saldanha
Member Secretary

Ref.No.DYPV/EC /382 / 2019

Date: 25 November 2019

After reviewing all documents IEC granted approval for the conduct of study and study documents. We approve the study to be conducted in its presented form.

Dr.M.S.Barthwal, Department of Respiratory medicine, Dr.D.Y.Patil Medical college Hospital & research centre Pimpri,Pune-18,being the principal investigator and any of the proposed study team members did not participate in the decision making process of the Ethics Committee and voting pertaining to this study. The Ethics Committee expects to be informed about the progress of the study, any SAE occurring in the course of the study, any changes in the protocol and patient information / informed consent /assent and asks to be providing a copy of the final report. I hereby certify that this committee is organized and operates according to ICH GCP Guidelines and schedule Y. At the time of the voting the required quorum was present.

Yours Sincerely,

Dr. (Brig) D Saldanha
Member Secretary
Dr.(Brig.) Daniel Saldanha
Member Secretary

Ethics Committee Dr.D.Y.Patil Vidyapeeth, Pune
Pimpri, PUNE - 18
